# How Environmental Factors Affect the Production of Guanidine Alkaloids by the Mediterranean Sponge *Crambe crambe*

**DOI:** 10.3390/md15060181

**Published:** 2017-06-16

**Authors:** Eva Ternon, Erica Perino, Renata Manconi, Roberto Pronzato, Olivier P. Thomas

**Affiliations:** 1 Université Côte d’Azur, CNRS, OCA, IRD, Géoazur, 250 rue Albert Einstein, 06560 Valbonne, France; 2 Dipartimento di Scienze della Terra, dell’Ambiente e della Vita, Università di Genova, Corso Europa 26, 16132 Genoa, Italy; ericaperino@yahoo.it (E.P.); pronzato@dipteris.unige.it (R.P.); 3 Dipartimento di Scienze della Natura e del Territorio, Università di Sassari, Via Muroni 25, 07100 Sassari, Italy; r.manconi@uniss.it; 4 Marine Biodiscovery, School of Chemistry, National University of Ireland Galway, University Road, H91 TK33 Galway, Ireland

**Keywords:** sponge, abiotic factors, specialized metabolome, metabolomics, sponge farming

## Abstract

Most marine sponges are known to produce a large array of low molecular-weight metabolites which have applications in the pharmaceutical industry. The production of so-called specialized metabolites may be closely related to environmental factors. In this context, assessing the contribution of factors like temperature, nutrients or light to the metabolomes of sponges provides relevant insights into their chemical ecology as well as the supply issue of natural sponge products. The sponge *Crambe crambe* was chosen as a model due to its high content of specialized metabolites belonging to polycyclic guanidine alkaloids (PGA). First results were obtained with field data of both wild and farmed specimens collected in two seasons and geographic areas of the North-Western Mediterranean. Then, further insights into factors responsible for changes in the metabolism were gained with sponges cultivated under controlled conditions in an aquarium. Comparative metabolomics showed a clear influence of the seasons and to a lesser extent of the geography while no effect of depth or farming was observed. Interestingly, sponge farming did not limit the production of PGA, while ex situ experiments did not show significant effects of several abiotic factors on the specialized metabolome at a one-month time scale. Some hypotheses were finally proposed to explain the very limited variations of PGA in *C. crambe* placed under different environmental conditions.

## 1. Introduction

Marine sponges are known to produce a large array of small molecules also called specialized metabolites that are likely to be involved in allelopathic interactions [1,2,3,4,5,6]. From a biotechnological point of view, such metabolites are frequently investigated, mostly for pharmaceutical applications [7,8,9]. Several biotechnological approaches have been developed for the large-scale production of these high value-added marine products which include in situ and ex situ farming, primmorph or cell culture [10,11]. Farming explants in situ has been considered as one of the most cost-effective ways to guarantee sufficient sustainable bioactive metabolites [12]. Since spatial and temporal variations in concentrations of major specialized metabolites have been documented previously [13,14,15], the ideal farming location that promotes their biosynthesis as well as the harvest season have to be assessed. The natural variations in the production of the specialized metabolites have been mostly explained by environmental and ecological factors like biotic interactions [16,17,18,19], abiotic factors [13,20,21,22] or more rarely, the organism’s life cycle [15,23] as well as physiological state [24,25]. Overall, the effects of abiotic factors on the production of specialized metabolites have produced contrasting results so far [20,26,27,28,29]. As highly effective filter feeders, marine sponges are able to take up a large range (<0.2–50 µm) of inorganic and organic particles from seawater [30]. Therefore, the environmental conditions, such as the seawater temperature or the availability of nutrients, are expected to influence the sponge metabolism, and assessment of their impact on the biosynthesis and concentrations of specialized metabolites is of high relevance in the context of sponge farming. 

In contrast to previous studies where a targeted analysis of a small number of identified compounds was performed, comparative metabolomics allows the monitoring of environmental changes using a broader range of metabolites [31]. Metabolomics refers to the set of low molecular-weight molecules (<1.5 kDa) biosynthesized by the cells in association with its metabolism, defined as the metabolome [32]. Providing a snapshot of the metabolic phenotype at a given time, metabolomics has the potential to determine biomarkers indicative of the effects of abiotic stressors such as natural and anthropogenic factors [33]. Therefore, the comparison of metabolomes for the investigation of organism-environmental interactions using high resolution analytical technology constitutes a powerful tool. In this study, we used a metabolomics approach using UHPLC-HRMS to assess the influence of seasonality and geographical distribution on the specialized metabolome of the Mediterranean encrusting sponge *Crambe crambe* (Porifera, Demospongiae) for wild populations and in situ farms. In a second set of experiments performed ex situ, we investigated the environmental factors likely to be responsible for the detected variations in situ (temperature, light, and nutrients availability).

The specialized metabolome of *C. crambe* has been well characterized during the past decades. It is composed of two families of polycyclic guanidine alkaloids (PGA, Supplemental information Figure 1); the crambescins (one or two cycles) and crambescidins (five cycles) that both exhibit substantial biological activities [34,35,36,37,38,39]. From *C. crambe*, several crambescidins (named from their molecular weight i.e., 800, 816 and 830) have been isolated and characterized [36,40,41] together with three sub-families of crambescins A–C differing in the presence of a pyrrolidine ring (A), a spiroaminal (B) and a linear 3-hydroxypropyl chain (C) [37,40,42]. We recently showed that crambescidins might form a chemical shield around the sponge that contributes to the sponge’s protection and ecological success [6]. Alteration of this chemical shield due to changes in environmental conditions will affect the allelopathic interactions that the sponge maintains with its habitat.

## 2. Materials and Methods

• Collection of wild and farmed sponges

**Figure 1:**
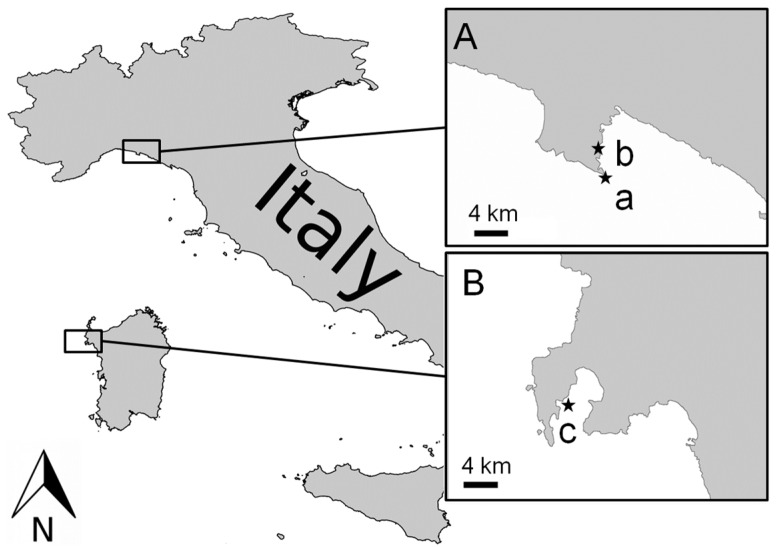
Collection sites of *Crambe crambe* in the Ligurian Sea (**A**) (a Punta del Faro, b Punta Pedale); and Sardinia Sea (**B**) (c Capo Caccia).

Triplicates of wild specimens of *C. crambe* were collected at different depths (5, 10 and 25 m) by SCUBA diving in the Ligurian Sea (Tigullio Gulf Punta del Faro 44°29′84.83″ N, 9°21′86.02″ E) in April (spring) and September 2013 (autumn) and in the Sardinia Sea (Porto Conte Bay, 40°35′43.69″ N, 8°12′31.44″ E) (Figure 1(A-a,B-c)) in April 2013 (spring). Autumn and spring correspond respectively to the assumed maximum and minimum production of guanidine alkaloids by *C. crambe* [17]. Farmed specimens of *C. crambe* were collected by SCUBA diving in the Ligurian Sea (Tigullio Gulf, Punta Pedale 44°19′15.35″ N, 9°12′57.00″ E) and in the Sardinia Sea (Tramariglio Bay, 40°35′32.60″ N, 8°10′11.72″ E) (Figure 1(A-b,B-c)) in April and September 2013. After collection, the sponges were immediately frozen at −20 °C, freeze-dried and maintained at −20 °C until extraction.

Mariculture of *C. crambe* was performed using the methodology USAMA^®^ [43] using underwater modular apparatus composed of different structures: (1) a stainless steel plant, composed of 1.5 × 1.5 m^2^ modules that can be combined in different ways (Supplementary Materials Figure S2A,B). Several pivots are used to settle, at equal distance, the ropes with the sponge explants (necklaces); (2) a PVC modular plant, made by rectangular or square forms (Supplementary Materials Figure S2C,D) anchored by ropes to structures. Pivots are inserted to anchor the sponge necklaces. The sponge explants were assembled in the ‘sponge necklaces’ [44]: (i) travertine tile method, composed of 10 cm × 10 cm travertine tiles anchored to the ropes and the rope drilled into the corner of rocky tablets. The sponge explants were pasted on each tile using a non-toxic underwater bi-component epoxy putty (SubCoat, Veneziani Yacht Systems, Italy) (Supplementary Materials Figure S2E); (ii) nylon mesh method, made of a posh-like net with 2 cm meshes fixed to ropes. The sponge explants were enclosed in the meshes (Supplementary Materials Figure S2F).

The abiotic sea parameters were obtained by the multiparameter probe ‘Idronaut’ in the Portofino Promontory area from 0 to 35 m (Povero pers. comm.). At both the Liguria and Sardinia sites, water temperatures were recorded by means of an underwater HOBO^®^ Data Logger (Onset, MA, USA) installed in the plants and in the wild. The temperature was recorded every 6 h. Monthly average temperature values were then considered.

• **Aquarium** **experiments**

*Crambe crambe* specimens were sampled in the bay of Villefranche-sur-mer (20 m depth along the rocky coast) in October 2013 (Temperature & Light experiment) and in January 2014 (Nutrient experiment). Specimens were carefully transferred to the Observatory of Villefranche-sur-mer and acclimated for one month in aquaria supplied by continuous seawater flow (2 L·min^−1^) pumped at a 5 m depth in the bay of Villefranche-sur-mer with a natural light/dark cycle as described previously [45]. Each sponge specimen was cut into small replicates of similar size (~5 cm^2^) in order to perform the two following sets of experiments: (1) “Temperature and Light experiment” and (2) “Nutrient experiment” that investigated the effects of variation in those environmental parameters on the specialized metabolism of *C. crambe*. Being collected from an encrusted rocky substrate, the specimens were carefully cut into different pieces with a hammer and a chisel to produce genetically identical biological replicates. 

### 2.1. Temperature and Light Experiment 

This first experiment was performed in November 2013, using a running seawater flow rate of 2 L·min^−1^. The five following treatments were implemented: (i) control (*T* = 16 °C; natural light cycle), (ii) light 24/24 h (300 lux), (iii) dark 24/24 h (0 lux), (iv) 14 °C, and (v) 20 °C. The collection of sponge specimens was performed after 0, 7 and 30 days, in each aquarium and in triplicate. Water temperature and light intensity were recorded in all aquaria by means of an underwater HOBO^®^ Data Logger (Onset, MA, USA). The probes were set to record temperature 2 times per day (every 12 h).

### 2.2. Nutrient Experiment

The second experiment was conducted in March 2014, using running seawater at a flow rate of 0.7 L·min^−1^. Five aquaria each containing 4 sponge replicates were set up: 2 control aquaria with only running seawater and 3 treatment aquaria with running seawater plus the addition of a nutrient mixture. The nutrient mixture was made up of MQ water and inorganic salts, i.e., phosphate (KH_2_PO_4_) and nitrate (NaNO_3_), and was brought to the three treated aquaria by a peristaltic pump at a flow rate of 1 mL·min^−1^ in order to reach a constant concentration of phosphate (PO_4_^3−^ = 2.5 µmol·L^−1^) and nitrate (NO_3_^−^ = 18 µmol·L^−1^). These concentrations were chosen as they are consistent with the anthropogenized site of Cortiou, close to the city of Marseille in the Northwestern Mediterranean Sea [46]. At T0 and after 5, 11, 18 and 24 days, sponge replicates were collected from each aquarium, and seawater was sampled from one control and one treated aquaria. Seawater samples were spiked with mercuric chloride and kept at +4 °C. Dissolved inorganic salts (PO_4_^3−^, NO_3_^−^, NO_2_^−^ and SiOH_4_) in seawater were then analyzed by a colorimetric method, using a Continuous Flow Automated Analyzer Technicon AutoAnalyzer II following a method developed previously [47]. For each set of experiments, sponge samples were flash-frozen using liquid nitrogen and maintained at −26 °C until chemical treatment.

• **Metabolomic** **analysis**

Methanol, acetonitrile and dichloromethane (Chromasolv^®^, analytical grade), formic acid (puriss. P.a. ~98%) and ammonium formate (LC-MS Ultra for UHPLC-MS) were purchased from Sigma Aldrich (Saint-Quentin Fallavier, France). Ultrapure water was prepared using a Milli-Q water system (Millipore Ltd., Billerica, MA, USA).

All sponge samples were freeze-dried and ground to obtain a dry powder which was extracted three times with a 15 mL mixture of MeOH/CH_2_Cl_2_ (1:1, *v*/*v*) in an ultrasonic bath (35 kHz) at room temperature. Due to the encrusting growth form of the sponge *C. crambe*, the rocky substrate was burnt at 550 °C for 24 h to obtain the sponge organic weight (Total weight − rock weight = sponge weight). Crude extracts were filtered through 8 µm paper filter (Whatman Grade 2V), dried using a rotary evaporator and further fractionated by solid phase extraction over a 500 mg C18 silica SPE cartridge (Phenomenex, Torrance, CA, USA) with a step gradient of H_2_O (20 mL), MeOH (20 mL), CH_2_Cl_2_ (20 mL). The methanol fraction was dried using an SPD111 SpeedVac (Thermosavant, Model RH12-28), concentrated to 10 mg·mL^−1^, and stored at −26 °C until further dilution prior to LC-MS analysis. 

*UHPLC-HRMS* *analysis*

Prior to the UHPLC-HRMS analysis, all samples were solubilized in 1 mL of MeOH and were diluted 1/1000. In addition, five Quality Control (QC) samples were prepared by combining 10 µL of each sample in a 2 mL vial and these pools were injected every seven samples to allow chromatogram alignment during data treatment. In-line UHPLC-UV-HRMS analysis was performed using a Dionex system Ultimate 3000 equipped with an autosampler and a Dionex Ultimate 3000 diode array detector (210 and 280 nm detection), connected to a qToF mass spectrometer with an electrospray ionization interface (Bruker Impact II). Mass spectra were recorded in the positive mode. UHPLC separation was achieved on an analytical Nucleodur PolarTec column (100 × 2 mm, 1.8 µm, Macherey Nagel) using a linear elution gradient of H_2_O/CH_3_CN/formic acid to which was added 10 mM of ammonium formate from 80:20:0.1 (*v*/*v*/*v*, isocratic from 0 to 2 min) to 40:60:0.1 (*v*/*v*/*v*, isocratic from 8 to 10 min), at a flow rate 0.45 mL·min^−1^ for a total of 14 min. The injected volume was set at 10 µL and detection at 280 nm. The mass spectrometer analyzer parameters were set as follows: nebulizer sheath gas, N_2_ (2.1 bar); dry gas, N_2_ (8 L/min); capillary temperature, 200 °C; capillary voltage, 2500 V; end plate offset, 500 V; collision gas, He; collision energy, 4 eV. Data were acquired in the 50 to 1200 *m/z* range. 

*UHPLC-HRMS data* *processing for untargeted analyses*

Following UHPLC-ESI-HRMS data acquisition, MS chromatograms (Base Peak chromatogram—BPC) were exported as line spectra and converted into the netCDF file format to process the data in centroid mode with XCMS [48] using R software (version 3.2.2.). The XCMS approach involved several steps necessary to generate a final matrix: (1) Peack picking (peakwidth = c(2, 20), ppm = 10) without threshold prefilter [49], (2) retention time correction (method = ”obiwarp”), (3) grouping (bw = 10, minfrac = 0.3, minsamp = 1), (4) Fillpeaks using a parameter of “noise level” that sets the minimum intensity for a centroid data point to be considered as part of a peak (noise level = 10^3^), and finally (5) report and data matrix generation (ions/Retention time × sample) that was exported using Microsoft Excel. Two successive filtering steps were applied to the matrix in R in order to suppress data of high analytical variability: (i) signal/noise ratio > 10 between variables matching in both extracts and blank samples (considers that ions are part of the noise if their mean intensity is less than 10-fold the blank value) and (ii) coefficient of variation in the intensity of the variables for the QC samples <20%. Each peak area was further normalized (according to the drift of equivalent ion of pooled samples), mean-centered (the mean ion intensity of all samples was subtracted from each sample ion intensity) and finally log-transformed to make individual features more comparable, using on-line MetaboAnalyst 3.0 [50]. Multivariate analysis was conducted (Principal components analysis (PCA) and Partial Least Squares-Discriminant Analysis (PLS-DA)) and PCA was further chosen since it gave the best discrimination. Very important peaks (VIPs) were determined according to the PCA loading plots.

*Metabolomic* *targeted analysis*

Chemical identification was performed only for compounds of interest (crambescins and crambescidins, Supplementary Materials Figure S2) that have been extensively described. The chromatogram obtained between 5.5 and 8 min of retention time was considered our metabolomic window, and the major compounds of this area were thoroughly identified by comparing their MS pattern with a standard (Supplementary Materials Table S1) [5,37]. Because several ion adducts can be detected for a single compound with electrospray ionization, the following mono- and di-charged adducts were also taken into account in the metabolite annotation: [M + H]^+^, [M + Na]^+^, [M + NH_4_]^+^, [M + 2H]^2+^/2, [M + 2NH_4_]^2+^/2. They are the most commonly observed in positive electrospray ionization mode with ammonium formate and formic acid as eluent modifiers. The theoretical *m/z* values for typical adduct species were compared with the experimental values to ensure the identification of the compounds (once per treatment and fraction and once for the QC samples). For spectra presenting multiple adducts, we summed the area of all adducts to obtain the total intensity corresponding to one compound. Analytical blanks confirmed that no memory effects or sample contamination skewed results. The concentration of the metabolites was assessed using a calibration with standards of crambescidin-816 and crambescin-A2-462, assuming all compounds of the same family had similar ionization potential. One-way ANOVA was performed on the data to investigate the statistical differences between conditions. 

## 3. Results

• In situ study with wild and farmed sponges

**Figure 2:**
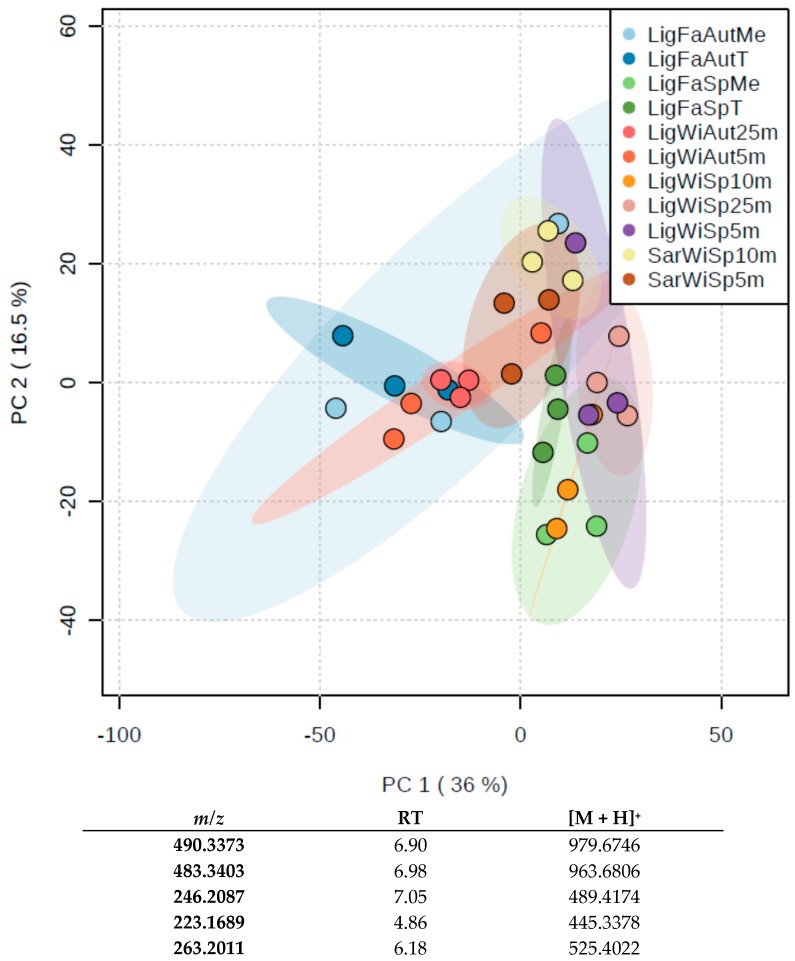
PCA plot obtained from the metabolomics study on wild (Wi) and farmed (Fa, Tile or Mesh protocols) samples harvested at different locations (Sar = Sardinia, Lig = Ligurian) in both autumn (Aut) and spring (Sp). The table lists the five most important ions contributing to the distribution of samples in the PCA plot.

To investigate depth, seasonal and geographic effects on the specialized metabolome of *C. crambe*, wild specimens were collected in the Ligurian Sea during both spring and autumn and in the Sardinia Sea during spring. A first metabolomics approach showed a significant effect of the season on the metabolome of wild specimens of *C. crambe* as revealed by the Principal Component Analysis (PCA) plot (Figure 2). Variations of the metabolic content in relation with geography or depth were not statistically significant. *Crambe crambe* was also farmed in the Ligurian Sea using both mesh and tiles protocols, and samples were collected in autumn and spring. The metabolomics approach shows that the specialized metabolome of specimens farmed are not significantly different from wild specimens, and the highest variability was attributed to seasonal changes (Figure 2). On the other hand, the farming protocols did not show significant differences in the specialized metabolome of *C. crambe*. Therefore, growing conditions and location have small impact on the content of the specialized metabolites of the sponge.

The five most contributive ions to this PCA were identified (Table S1) using full scan chromatograms for validation. Interestingly, most of the ions eluted between 4.8 and 7 min, corresponding to compounds slightly more polar than crambescins and crambescidins. None of these compounds were assigned to known metabolites. Therefore, we concluded that the variability of the metabolic content is not driven by the major metabolites (i.e., crambescins and crambescidins) but rather by minor metabolites. 

**Figure 3:**
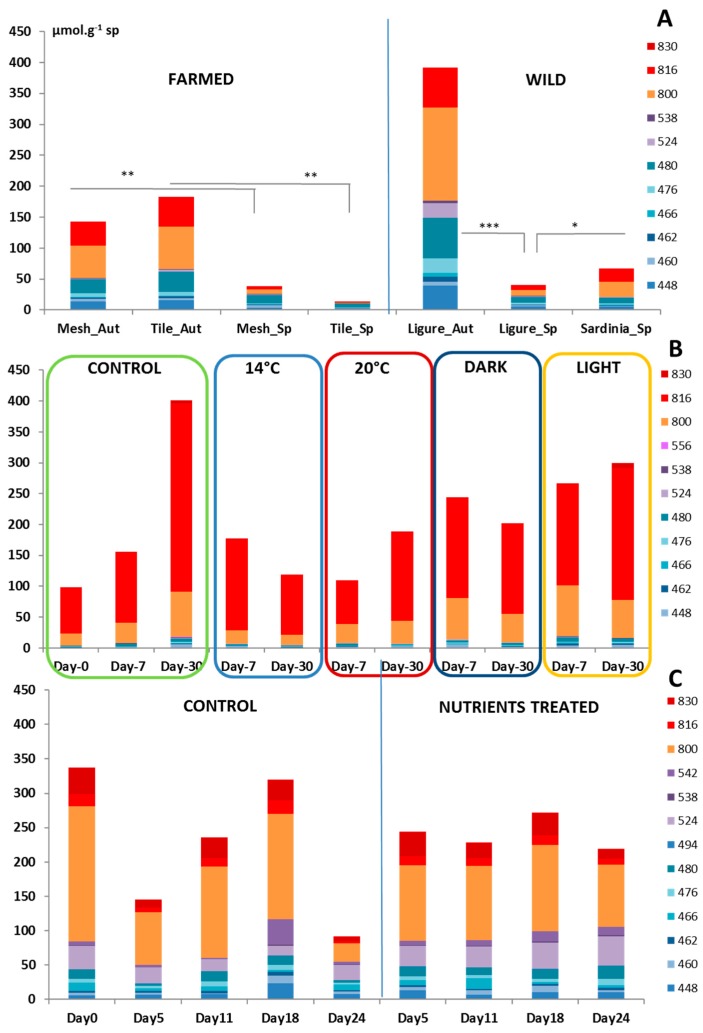
Concentrations of crambescidins and crambescins in *C. crambe* in µmol·g^−1^ sp obtained from the targeted metabolomics study for (**A**) wild and farmed specimens, (**B**) Temperature and Light experiment, and (**C**) Nutrient experiment (*** *p* < 5 × 10^−4^; ** *p* < 0.005; * *p* < 0.05, one-way ANOVA).

To better investigate the variations in the concentrations of the two major families of specialized metabolites, the crambescins and crambescidins, a targeted metabolomics analysis was undertaken. From this study, we outlined a lower concentration of both families during spring compared to autumn (39 against 391 µmol·g^−1^ sp, *p* = 1.55 × 10^−6^ ***, Ligurian samples; Figure 3A). The concentration of specialized metabolites in autumn was supported by both the crambescin and crambescidin families (up to 178 and 215 µmol·g^−1^ sp, respectively). A similar seasonal trend was shared by farmed specimens for both the crambescins and crambescidins families (Figure 3A). Wild and farmed samples showed almost significant (*p* = 0.0657) differences for both families of compounds; these differences being mostly supported by samples harvested in autumn (93 against 204 µmol·g^−1^ sp for the crambescins and 169 against 284 µmol·g^−1^ sp for the crambescidins). 

The spatial location of *C. crambe* slightly impacted the content of crambescidins since their concentrations in Sardinia were statistically higher than those in the Ligurian Sea (45 µmol·g^−1^ sp, *p* = 0.0338 *), a result mostly supported by the concentrations of crambescidins. It is noteworthy that the crambescins did not show any significant difference between the two regions (23 and 22 µmol·g^−1^ sp in Liguria and Sardinia, respectively).

• Ex situ experiments

Some experiments were also conducted in aquaria to study the variability of the specialized metabolome of *C. crambe* when submitted to diverse environmental factors. 

### 3.1. Temperature and Light Experiments

Although we attempted to keep fixed conditions of temperature in all aquaria, this was made difficult by an open running seawater system that is affected by natural variations. The temperature of the control aquaria naturally fluctuated between 15 and 17 °C, and a maximum of one-degree variation was recorded for both the controlled “14 °C” (between 13.5 °C and 14.5 °C) and the “20 °C” (between 19.5 °C and 20.5 °C) experiments. Light intensity was set at 300 Lux (~1200 W/m^2^) during the 12 h of a light-dark cycle in the temperature experiment. For the light experiments, the same cycle was maintained for the control experiment, and the results of the metabolomic analyses were compared with a permanent light experiment and permanent dark experiment. 

**Figure 4:**
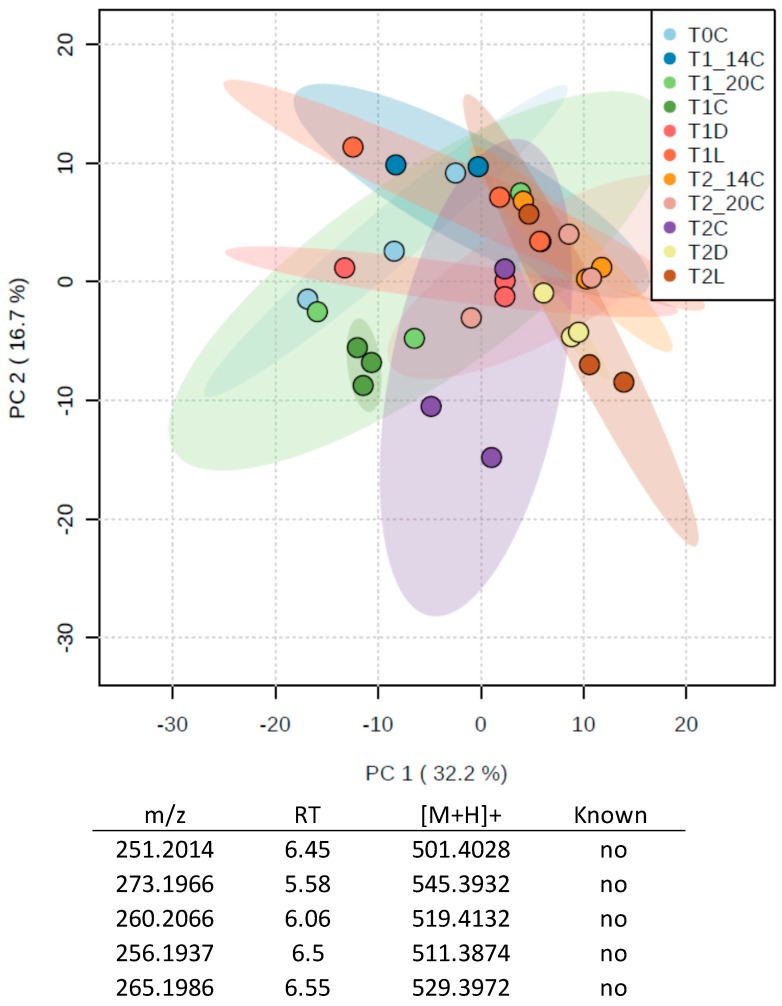
PCA plot obtained from an untargeted metabolomics study of the temperature/light experiment. C = control, D = Dark, L = Light, 14 °C = Temperature 14 °C and 20 °C = Temperature 20 °C. The table shows the five most important peaks that contribute to the distribution of samples on the PCA plot, along with their retention time.

A PCA plot revealed no clear difference between control, temperature and light experiments (Figure 4). It is noteworthy that the inter-sample variability within a treatment increased with the experimental time as shown by the distribution of the replicates. To be noted, the five most contributive ions to the PCA presented mid-polarity, very close to the PGA (5.5 < RT < 6.5), but were not assigned to any known metabolites.

The targeted metabolomics study confirmed the absence of statistically significant differences in the concentrations of the crambescins and crambescidins of *C. crambe* specimens across the treatments. The summed concentration of the major specialized metabolites was very close to the one measured in samples harvested in the field, although the proportion of crambescidin represented up to 50–70% of the total content (Figure 3B). Differences between treatments, for instance, the concentration of crambescidins in control at T2 (increase by a factor 2.7), were mainly driven by inter-individual variability. 

### 3.2. Nutrient Experiment

#### 3.2.1. Nutrient Concentrations

Although only nitrate and phosphate were added to the aquaria, four inorganic salts were measured in the seawater during the experiment: nitrate, nitrite, phosphate and silicate (SI, Figure S3). No difference in the concentration of non-added salts was noticeable between control and treated aquaria; those salts being most probably controlled by the sponge. The nutrient mixture was efficiently added to the treated aquarium as shown by the nitrate and phosphate concentrations (23 and 3 µmol·L^−1^ at T4, respectively). However, attempted homogeneous concentrations in treated aquaria were only reached at day 18 after important variations (PO_4_^3−^: 0.7–1.5 µmol·L^−1^; NO_3_^−^: 6–13 µmol·L^−1^). This instability may be explained by the technical issues encountered during the delivery of the nutrient mixture by the peristaltic pump. 

#### 3.2.2. Metabolomics Study

**Figure 5:**
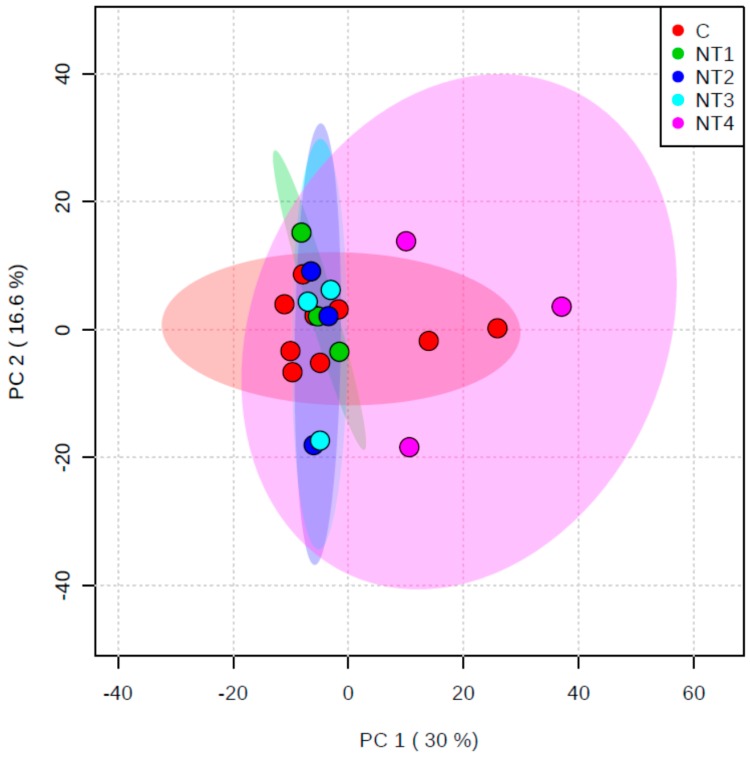

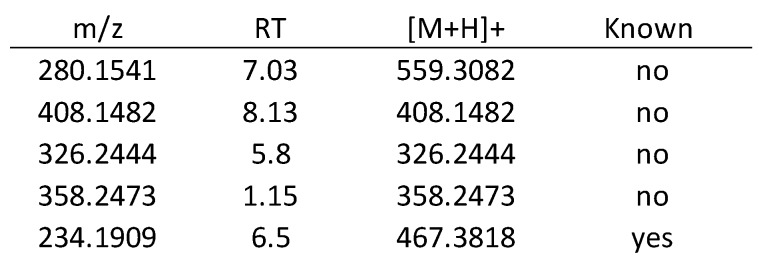
PCA plot obtained from an untargeted metabolomics study of the nutrients. C = control and N = nutrient-treated samples and triplicates of biological samples are represented by a colored point. The table shows the five most important ions contributing to explain the sample distribution on the PCA plot along with their retention time and their expected [M + H]^+^.

The distribution of the samples on the PCA showed no clear difference in the specialized metabolome of control and nutrient-treated samples (Figure 5). However, a change in the metabolome at T4 seemed to be initiated and should be considered. It is worth noticing that the most contributive ions differed in this experiment from the previous one. In this case, we were able to identify crambescin C1 with a lower concentration at T4 in nutrient-treated samples. 

The targeted metabolomics analysis showed that the differences observed between control and nutrient-treated samples were not statistically different for the major metabolites (Figure 3C). The concentrations measured in this experiment were very close to those measured in in situ samples. 

## 4. Discussion

The Mediterranean Sea is considered an oligotrophic region where spatial distribution of biogeochemical elements [51] as well as plankton species [52] is dependent on meso-scale circulation of water masses. Variations of such biotic and abiotic parameters might contribute to variations in the production of the specialized metabolites in common organisms like sponges. This aspect is of interest as sponges are recognized as important producers of metabolites with pharmaceutical interest. We selected the Mediterranean sponge *C. crambe* as a model due to the presence of a high quantity of bioactive alkaloids named Polycyclic Guanidinic Alkaloids from the crambescin and crambescidin families.

In order to assess the variability, specimens of both wild and farmed *Crambe crambe* sponges were harvested from two distinct Mediterranean sub-basins (Ligurian Sea and Sardinia Sea), located from ~500 kilometers along a latitudinal cline (44°18′ N vs. 40°35′ N, respectively), thus differing by the intensity of the solar radiation they receive as well as by their hydrological characteristics (temperature, salinity) and primary productivity [53]. The production of specialized metabolites in wild specimens was shown to be very similar between the two sampled locations (Figure 2). Therefore, the role played by environmental parameters in the concentration of specialized metabolites is limited in this case. This limited intraspecific metabolic variability observed between the two locations might be related to the genetic diversity as previously suggested [1,33,54,55,56,57]. In accordance with previous studies [13,14,15,20,23,58], a marked seasonal trend of the metabolic content showed a maximum in autumn and a minimum in spring (specimens of the Ligurian Sea, Figure 2). Interestingly, the same seasonal pattern was observed in both wild and farmed specimens cultured in this area, suggesting a role of internal physiological factors as main drivers of the variations of the specialized metabolism. Such factors could either be growth [21,24,25] or reproduction [57,58]. It has been often hypothesized that the release of larvae in late summer signals the end of the energy investment in reproduction, allowing a switch towards an enhanced production of specialized metabolites during autumn and throughout the winter [17]. This trade-off between the reproduction and the production of specialized metabolites was hypothesized by [24] and [17] for *C. crambe*. Our data set also reveals a significant difference in the metabolic content between wild and farmed specimens in autumn (Figure 2), with higher concentration of the toxic crambescidin family in wild sponges (Figure 3A). Thus, a combination of internal factors acting together with biotic factors such as competition for space [1,16,18,19] may also explain the seasonal variability of the production of the specialized metabolites. Abiotic factors such as temperature or nutrient availability might trigger other processes like sponge reproduction or other constraints of primary metabolism [25,58,59], rather than directly affecting the specialized metabolome. 

The metabolomics approach allowed encompassing of the variability of the whole set of specialized metabolites produced by *C. crambe* with both the minor and the major metabolites. It is interesting to note that none of the major specialized metabolites belong to the five most contributive ions. Minor specialized metabolites supported the variability of the metabolic content. However, since the seasonal and geographical trends of the major specialized metabolites revealed by the targeted analysis are very close to those observed for the minor metabolites, the variation of both contents is most likely closely related. This observation supports the idea of a combined contribution of both some associated bacteria and their host sponge cells. Indeed, these results are in accordance with relatively fast changes in the microbial production of key intermediate minor compounds under changing environmental conditions. However, the production of the major compounds PGA would be slower as they would require some key enzymes potentially present in sponge cells. 

**Figure 6:**
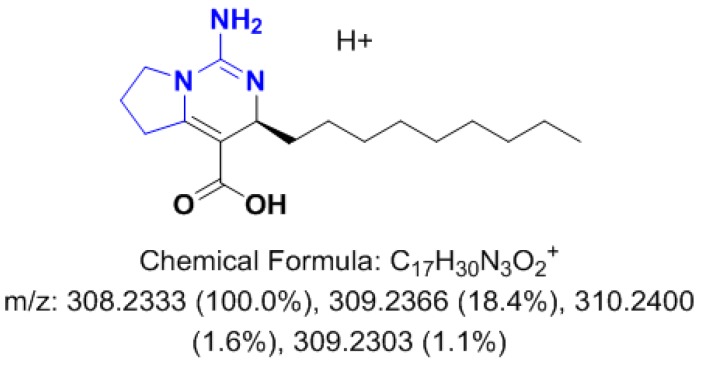
Proposition of a biosynthetic intermediate for crambescin A1.

Our assumptions of the minor role played by abiotic factors for the analysis of the variability of the metabolic content was confirmed by the two sets of experiments conducted in aquaria, where no relevant effects of light, temperature or nutrient availability on the whole specialized metabolome were observed (Figure 3B,C, Figure 4 and Figure 6). The absence of obvious links between the light intensity, the seawater temperature and the specialized metabolome is consistent with previous studies conducted either in situ [15,20,26,29] or in aquaria [28]. Although no relationship between the nutrient availability and the content of the specialized metabolism was demonstrated, DIN (dissolved inorganic nitrogen) consumption is known to be a key mechanism of nutrient acquisition in temperate sponges [60]. Since most, if not all, specialized metabolites of the sponge *C. crambe* belong to the PGA, an increase in DIN concentrations could have been expected to enhance their biosynthesis. The relationship between nutrient availability and specialized metabolites is believed to be more subtle than single uptake and use, especially since there is increasing evidence that sponge micro-symbionts are the first to consume the nutrients [61,62,63]. Indeed, some sponges were shown to obtain a significant portion of their nutrients from the bacterial symbionts themselves [64]. Likewise, symbionts may somehow be involved in the biosynthesis of specialized metabolites in several sponge species. If we recently observed that crambescins and crambescidins are stored in sponge cells [6], their biosynthesis could be at least be partly inferred to be related to some microbial symbionts. Betaproteobacteria, recently identified as major symbiont in *C. crambe*, contain a long-chain fatty acid ligase [65] that is likely to be involved in the biosynthesis of the upper alkyl chain of the crambescins [5]. A biosynthetic pathway for both families of compounds, implying several biosynthetic intermediates, e.g., the crambescin A1 carboxylic acid intermediate, was proposed (Figure 6). The corresponding [M + H]^+^ = 308.2347 was found after extraction of the base peak chromatogram within the same RT (6.14 min) of the crambescins A1 (462). Although variations in the concentration of this assumed intermediate was found across the seasons, following the trend observed for crambescin A (data not shown), the lack of clear identification [66] does not allow thorough conclusions. Additions of DIP and DIN could have eventually stimulated the production of first biosynthetic links through microbial use. 

Previous observations allowed us to propose a hypothesis for the low variations in the PGA concentrations under different experimental conditions. Indeed, the major PGA are likely to be stored at high concentration in spherulous cells [6], causing changes in the absolute concentrations of these compounds that are relatively non-significant over a short-term period. Small inputs of compounds in a large pool of stored ones will not strongly affect their final concentrations. We therefore expect that longer term experiments (months) would enable a clearer view of changes in the overall concentrations of PGA, but their stability at a shorter time (weeks) is remarkable. This storage buffering effect would also comprise a long-term defense strategy, especially against pathogenic microbes. 

## 5. Conclusions

The combination of the untargeted and targeted metabolomics approaches revealed that the specialized metabolome of *C. crambe* varies in two periods of the year, and those variations are not related to abiotic factors (temperature, light and nutrients). Biotic factors, like the reproduction cycle, but also additional abiotic factors other than light, temperature or nutrients, may contribute to the seasonal variability in the metabolome.

Ex situ experiments confirmed the limited impact of abiotic factors on the metabolic content and revealed stability in the concentrations of the major specialized metabolites at a short time scale. We assume that this stability could be related to a buffering effect due to the storage of compounds in spherulous cells, which would mitigate the metabolic changes. 

In the context of sponge-based biotechnologies, farming or ex situ cultures of *C. crambe* did not improve the biosynthesis of its specialized metabolites but did not significantly lower it. Since in situ cultivation of *C. crambe* by standardized methodology in sea-based farming structures enhances its mean growth (reaches around ten times the initial volume per year [44]; Manconi, pers. obs.), an increase in the specialized metabolite content over the years could be expected, promoting this cultivation protocol as a sustainable way to harvest specialized metabolites with low environmental impacts. Combining this process with our sustainable extraction of PGA from *C. crambe* [45] may represent a highly efficient process for the medium-scale production of PGA.

## Supplementary Materials

The following are available online at www.mdpi.com/1660-3397/15/6/181/s1.
