# Supplementary material for: How Environmental Factors Affect the Production of Guanidine Alkaloids by the Mediterranean Sponge Crambe crambe
Source: Mar Drugs. 2017 Jun 16;15(6):181. doi: 10.3390/md15060181 (PMC5484131; doi:10.3390/md15060181)
Supplement: Supplementary file 1 [file marinedrugs-15-00181-s001.pdf]

Supplemental Information

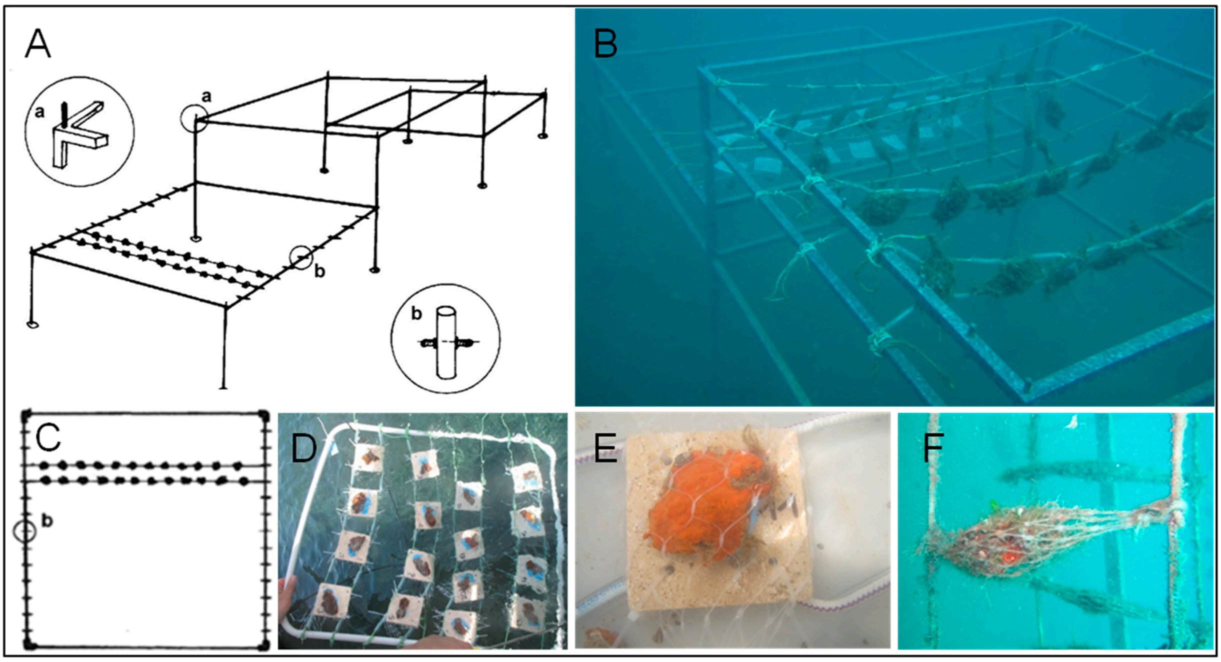

Figure 1. *Crambe crambe* farming structures - A and B: stainless steel plant, C and D: PVC structure, ‘sponge necklace’ methods E: Travertine Tile, F: Mesh.

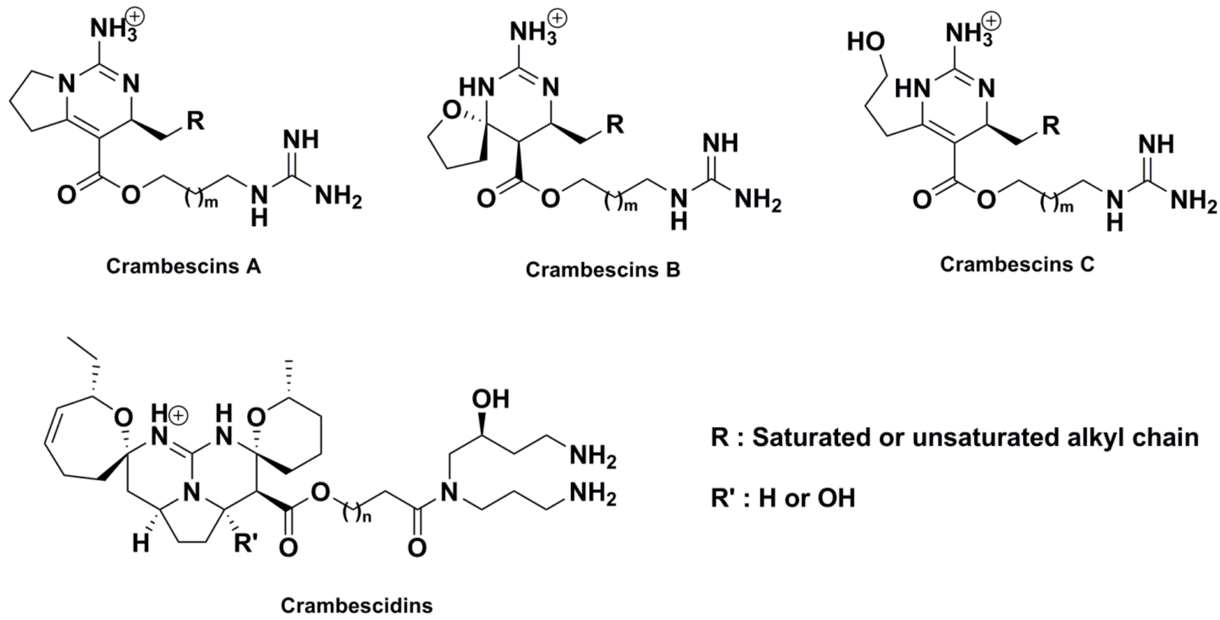

Figure S 2. Structure of the members of the two chemical families crambescins and crambescidins

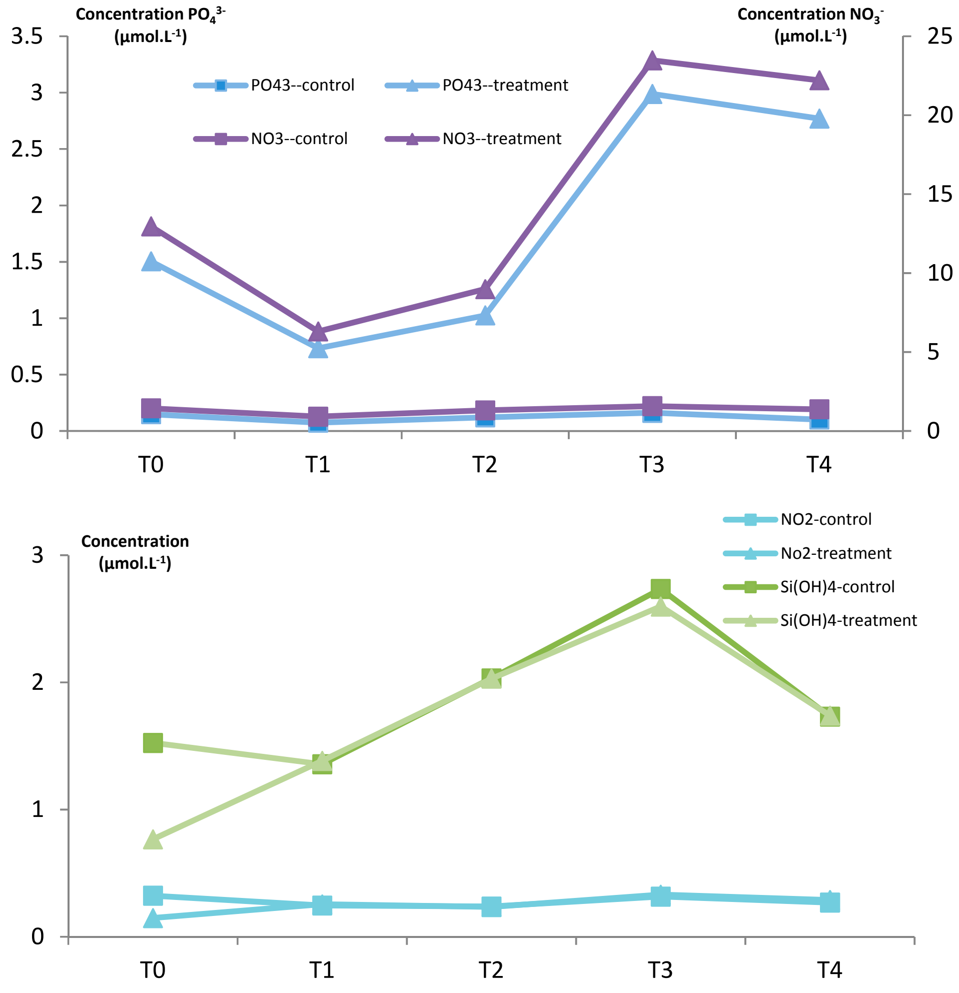

Figure 3. Evolution of the concentration (µmol.L^{-1}) of the inorganic nutrients (PO_4^{3-}, NO_3^-, NO_2^- and $Si(OH)_4$) in the control (square) and the treated (triangle) aquaria during the nutrients experiment.

Table 1. Theoretical m/z and associated molecular formula for quantified PGA from *C. crambe* (derived from ref.37).

		$[M+H]^+$	Empirical formula
**Crambescin**	**A2**	449.36017	$C_{24}H_{45}N_6O_2$
	**Didehydro A1**	461.35976	$C_{25}H_{44}N_6O_2$
	**A2**	463.37552	$C_{25}H_{47}N_6O_2$
	**B1 / C1**	467.37051 / 467.37027	$C_{24}H_{47}N_6O_3$
	**A2**	477.39197	$C_{24}H_{49}N_6O_2$
	**B1 / C1**	481.38629 /481.3863	$C_{25}H_{49}N_6O_3$
	**B1 / C1**	495.40207 / 495.40182	$C_{26}H_{51}N_6O_3$
	**A3**	525.3915	$C_{30}H_{49}N_6O_2$
	**A3**	539.4081	$C_{31}H_{51}N_6O_2$
	**B3 / C3**	543.4016	$C_{30}H_{51}N_6O_3$
	**B3 / C3**	557.4167	$C_{31}H_{56}N_6O_2$
	**Didehydro C3**	555.4024	$C_{31}H_{51}N_6O_3$
**Crambescidin**	**800**	801.6242	$C_{45}H_{82}N_6O_6$
	**816**	817.6151	$C_{45}H_{81}N_6O_7$
	**830**	831.63	$C_{46}H_{83}N_6O_7$
